# A Study of Null Effects for the Use of Transcranial Direct Current Stimulation (tDCS) in Adults With and Without Reading Impairment

**DOI:** 10.1162/nol_a_00020

**Published:** 2020-10-01

**Authors:** Jacqueline Cummine, Miya Villarena, Taylor Onysyk, Joseph T. Devlin

**Affiliations:** Communication Sciences and Disorders, University of Alberta, Edmonton, Canada; Neuroscience and Mental Health Institute, University of Alberta, Edmonton, Canada; Neuroscience and Mental Health Institute, University of Alberta, Edmonton, Canada; Communication Sciences and Disorders, University of Alberta, Edmonton, Canada; Experimental Psychology, University College London, London, UK

**Keywords:** brain modulation, reading, dyslexia, tDCS

## Abstract

There is evidence to support the hypothesis that the delivery of anodal transcranial direct current stimulation (tDCS) to the left temporoparietal junction can enhance performance on reading speed and reading accuracy (Costanzo et al., 2016b; Heth & Lavidor, 2015). Here, we explored whether we could demonstrate similar effects in adults with and without reading impairments. Method: Adults with (*N* = 33) and without (*N* = 29) reading impairment were randomly assigned to anodal or sham stimulation conditions. All individuals underwent a battery of reading assessments pre and post stimulation. The stimulation session involved 15 min of anodal/sham stimulation over the left temporoparietal junction while concurrently completing a computerized nonword segmentation task known to activate the temporoparietal junction. Results: There were no conclusive findings that anodal stimulation impacted reading performance for skilled or impaired readers. Conclusions: While tDCS may provide useful gains on reading performance in the paediatric population, much more work is needed to establish the parameters under which such findings would transfer to adult populations. The documentation, reporting, and interpreting of null effects of tDCS are immensely important to a field that is growing exponentially with much uncertainty.

## INTRODUCTION

Literacy skills are characterized by performance in reading, spelling, and/or related tasks (Peterson & Pennington, 2015; Tunmer & Greaney, 2010; Eden & Zeffiro, 1998). Literacy impairment (e.g., developmental dyslexia) can have a profound impact on an individuals’ academic achievement, career opportunities, mental well-being, and social life, particularly in today’s society where social media platforms are extensive and rely heavily on written communication (e.g., e-mail, Facebook, Twitter, etc.). Individuals with impaired literacy skills experience decreased opportunities for employment, overall lower paying jobs, decreased success in educational environments, and increased mental health issues (i.e., depression and anxiety), just to name a few. To make matters worse, there are no cures for literacy-based learning disabilities. Many remediation approaches have been proposed that target specific skill-based training (i.e., letter-to-sound mapping, phonemic decoding, fluency); however, these are best implemented in childhood when the skills are still developing. They are not always effective, with vast variability in literacy profiles, responders -versus non-responders, and often, these children continue to have persistent literacy difficulties through adulthood. Overall, these findings underscore the need to explore novel approaches, such as neuromodulation, to remediating reading difficulties in adulthood.

### Neuromodulation

Transcranial direct current stimulation (tDCS) is a noninvasive neurostimulation technique that involves direct current delivery to the level of the scalp (Filmer, Dux, & Mattingley, 2014; Monti et al., 2013; Stagg & Nitsche, 2011). Negative cathodal stimulation hyperpolarizes neurons whereas positive anodal stimulation depolarizes neurons (Thair, Holloway, Newport, & Smith, 2017). Placement of the positive and negative electrodes into various montages is purposeful to upregulate and/or downregulate activity in specific brain regions. The recommended current delivery range is between 1–2 mA, with no adverse or harmful effects being documented except for mild itching or tingling sensations (Brunoni et al., 2012; Nitsche et al., 2004). Direct current delivery (which varies in current strength, measured in milli-amperes, mA) can be described in terms of charge density (C/cm^2^) and duration of current application, both of which elicit changes during and after stimulation (Stagg & Nitsche, 2011). The changes during stimulation are a result of subthreshold alterations in neuron resting membrane potential; a dose of anodal stimulation elevates the resting membrane potential, causing neurons to be more easily excited, whereas cathodal stimulation brings the neurons to a more negative resting potential, increasing the difficulty for excitation (Filmer et al., 2014; Nitsche & Paulus, 2001; Thair et al., 2017). The evidence for this comes from combined tDCS plus transcranial magnetic stimulation studies that measure induced motor evoked potentials (Nitsche & Paulus, 2001) and pharmacological experiments. For example, Nitsche et al. (2003) showed that blocking calcium and sodium ion channels with flunarizine and carbamezipine was able to attenuate the effects of anodal stimulation, highlighting the importance of ion channels in the elevation of the resting membrane potential and subsequent anodal effects.

### Neuromodulation and Reading

Within the paediatric literature, a handful of researchers have provided supportive findings for the use of tDCS in the remediation of reading difficulties (Costanzo et al., 2016a, 2016b, 2019; Rahimi et al., 2019; Rios et al., 2018). For example, Costanzo et al. (2016b) tested the effects of single session tDCS (20 min, 0.04 mA/cm^2^ current density) on reading performance with different montages: concurrent anodal left temporoparietal junction stimulation with right homologue cathodal stimulation, as well as the reverse (cathodal left hemisphere, anodal right). Overall, they found a reduction in reading errors on a 400-word Italian text during left anodal/right cathodal stimulation, but diminished accuracy when the right anodal/left cathodal condition was administered. Such effects have also been reported for nonword and word accuracy (Rios et al., 2018), and auditory processing speed (Rahimi et al., 2019) with single anodal stimulation conditions over the left temporal region.

While adult reading studies with tDCS are often varied and conflicting (Heth & Lavidor, 2015; Thomson, Doruk, Mascio, Fregni, & Cerruti, 2015; Turkeltaub et al., 2012; Younger, Randazzo Wagner, & Booth, 2016; see also Minarik et al., 2016, for a discussion of how power in tDCS studies translates to mixed findings), the significant effects associated with stimulation warrant further investigation. For example, Turkeltaub et al.’s (2012) study on left lateralization of reading efficiency reported that delivering anodal stimulation (via 5 × 5 cm positive electrode at 1.5 mA, 20-min stimulation) to the left temporoparietal junction and cathodal stimulation to the right homologue was able to improve performance on reading both words and nonwords. In contrast, it appears that right and not left temporoparietal junction stimulation improves letter-to-sound mapping in adults (Thomson et al., 2015). To further complicate matters, it appears that left hemisphere stimulation can be detrimental to performance in some cases. For instance, Younger et al. (2016) reported anodal stimulation of the left inferior parietal lobule (via 5 × 5 cm positive electrode at 1.5 mA, 20-min stimulation) enhanced single word reading efficiency but impaired rhyme judgment. As such, additional work is still needed—particularly approaches that take into account the recommendations outlined in Minarik et al. (2016) with respect to power and sample size—to answer the question: Does anodal tDCS over the left temporoparietal junction enhance reading performance in adults?

Given the promising effects of neuromodulation on reading performance in the paediatric population, perhaps the mixed findings in the adult population are due to a reduced capacity for neural plasticity (see Cancer & Antonietti, 2018, for a review of nine papers that explore tDCS effects on reading performance). As noted in several papers (Costanzo et al., 2016a, 2019; see also Younger & Booth, 2018, for an example of neuromodulation plus reading training in adults), one possible way of boosting the effects of neuromodulation is to apply simultaneous stimulation, whereby the stimulation is paired with a task that utilizes the same brain region that is being targeted for stimulation and the subsequent behavioural performance. From neuroimaging, we have evidence that the temporoparietal junction (consisting of the posterior superior temporal gyrus, the inferior supramarginal gyrus, and the angular gyrus) is particularly sensitive to letter-to-sound mapping, among other things (Ramus, 2004). For individuals with reading impairments, the temporoparietal junction has been identified as underactivated by fMRI (Eden et al., 2004; Richlan, Kronbichler, & Wimmer, 2009; Shaywitz et al., 2002; Eden & Zeffiro, 1998), positron emission tomography (Rumsey et al., 1992), and MEG (Breier et al., 2003). On a structural level (i.e., diffusion tensor imaging), it has been reported that white matter tracts underlying the left temporoparietal junction have decreased structural integrity compared to typical readers (Klingberg et al., 2000; Deutsch et al., 2005; Hoeft et al., 2011; Rimrodt, Peterson, Denckla, Kaufmann, & Cutting, 2010; Niogi & McCandliss, 2006).

In the paediatric literature, we know that individuals with reading impairments often have deficits in letter-to-sound mapping (Navas, Ferraz, & Borges, 2014; Ramus, 2014; Snowling, 1981) and manipulation of the small sound units that comprise a word (e.g., phonemic awareness, reading of nonwords such as “yeighb”; Kochnower, Richardson, & DiBenedetto, 1983; Rack, Snowling, & Olson, 1992; Snowling, 1981). Previous research has shown that training for certain aspects of reading by segmenting words into small units of sound can improve future reading performance (Alexander & Slinger-Constant, 2004; Eden et al., 2004; Younger & Booth, 2018). The joint effects of neuromodulation plus skill-based reading training was recently explored by Costanzo et al., (2016a, 2019) in children with the following protocol: 3 sessions per week for 6 weeks, at 20 min per session, with anode on left parietotemporal and cathode on right parietotemporal regions, and current density 0.04mA/cm^2^. In this work, the authors showed that sessions of cognitive behavioural training, which included overt reading speed and covert phonics, alongside anodal stimulation were able to improve nonword reading speed and low-frequency word accuracy in Italian children with and without reading impairment. Those administered the sham condition saw no improvement overall, whereas those who received the anodal condition displayed improvements at both 1-month and 6-month timepoints, implicating a lasting beneficial effect of tDCS delivery alongside training. The lasting after-effects of tDCS have been documented (Brunoni et al., 2012; Monte-Silva et al., 2013; Nitsche & Paulus, 2001; Bindman, Lippold, & Redfearn, 1964) and are attributed to mechanisms of synaptic plasticity similar to long-term potentiation (see Bear & Malenka, 1994; Bliss & Lømo, 1973; Liebetanz, Nitsche, Tergau, & Paulus, 2002; Monte-Silva et al., 2013; Nitsche et al., 2003; Stagg & Nitsche, 2011, for discussions on molecular changes via NMDA receptor antagonist dextromethropane, agonist d-cycloserine, and Ca2+).

In a recent study by Younger and Booth (2018), adults underwent training to learn a new orthography and either had anodal stimulation over the left temporoparietal junction (5 cm × 5 cm, 1.5 mA, 20-min, 3 sessions) or sham stimulation. Similar to previous work, the authors found that stimulation benefitted adults with lower reading skills to a greater degree than adults with higher reading skills. Notably, Younger and Booth (2018) did not deliver stimulation at the same time as training, and thus it remains unknown the extent to which there are potential benefits of simultaneous neuromodulation and training in adults with and without reading difficulties.

### The Current Study

There is some evidence to support the hypothesis that (1) the delivery of tDCS to the left temporoparietal junction can enhance performance on reading speed and reading accuracy (Costanzo et al., 2016b; Heth & Lavidor, 2015) and (2) simultaneous tDCS and training can improve reading performance (Costanzo et al., 2016a, 2019; Alexander & Slinger-Constant, 2004; Eden et al., 2004). Here, we aim to explore the impact of simultaneous neuromodulation and training on reading performance in adults with and without reading impairments. Based on the recent review by Cancer and Antonietti (2018), we anticipate that the training, in general, would enhance reading performance (i.e., accuracy and reading speed) for individuals with impaired reading ability. We further anticipate that stimulation plus training would improve reading speed in both groups.

## MATERIALS AND METHODS

### Participants

Adults (*N* = 62; age >18) were recruited to take part in the study. Individuals were classified as having a reading impairment based on the following criteria: (1) they self-identified as having previous reading and learning difficulties and (2) at least one overt reading score (using the Word Identification and Word Attack standardized reading tests; Woodcock & Johnson, 1989) that falls 2 *SD* below the mean of the skilled adult readers. The subjects that were recruited were all over 18 (*M* = 22.125; *SD* = 3.442; 5 females), had no previous history of stroke, migraines, seizures, and epilepsy, and had no existing comorbidities with attention deficit hyperactive disorder. All subjects were right-handed, had normal or corrected normal vision, and were proficient in English. Two participants were removed due to equipment failure (i.e., no dependent measures recorded). The final groups were Impaired (*N* = 32; Females = 29) and Skilled (*N* = 28; Females = 20; see Table 1 Descriptive Statistics). Consent was obtained according to the Declaration of Helsinki (2013, https://www.wma.net/policies-post/wma-declaration-of-helsinki-ethical-principles-for-medical-research-involving-human-subjects/) and the experiment was performed in compliance with the relevant laws and institutional guidelines and was approved by the Health Research Ethics Board at the University of Alberta. All participants were paid a small honorarium.

**Table 1.** Descriptive statistics

	Age	Mean school years	Word identification	Word attack	Reading history questionnaire
Skilled	23.1 (4.4)	16.29 (2.4)	102.75 (1.3)	43.52 (1.4)	0.16 (0.17)
Impaired	22.2 (4.1)	15.55 (2.4)	98.85 (2.2)	41.09 (2.5)	0.28 (0.16)
*p* value	0.413	0.243	*<0.001	*<0.001	*0.007

*Note*. Mean (*SD*) and group differences. *Significant difference (*p* < 0.05) between groups using an independent samples *t* test.

### Materials

Several tasks were chosen based on previous work on tDCS plus reading (Cancer & Antonietti, 2018; Turkeltaub et al., 2012; Younger et al., 2016; Younger & Booth, 2018). Specifically, reading efficiency (i.e., timed tasks that require participants to overtly generate single words/nonwords as quickly and accurately as possible) have been shown to be positively impacted by tDCS in adults (Turkeltaub et al., 2012; Younger et al., 2016). In addition, given the single session, single montage nature of the current study, we also opted to include an additional efficiency task that removed the reading component and just measured fluency (i.e., timed tasks that require participants to overtly generate single letters/digits as quickly and accurately as possible; Younger et al., 2016). Notably, the latter task is highly related to and predictive of reading ability and disability and may also be susceptible to training and/or stimulation changes in a single session.

#### Overt reading task

Two lists (40 words in each) for each stimulus type (regular words, nonwords, and pseudohomophones) were prepared and randomized for each participant, with one as the pre-stimulation task and the other as the post-stimulation task. Words were taken from the English Lexicon Project (Balota et al., 2007; mean length = 4.2, mean log frequency HAL = 8.3; mean orthographic neighborhood = 8.4; mean phonological neighborhood = 17.4; mean number of phonemes = 3.5; mean bigram = 1,420), while nonwords (mean length = 5.3; mean orthographic neighborhood = 1.6; mean bigram = 1,211) and pseudohomophones (mean length = 5.2; mean orthographic neighbourhood = 1.8; mean bigram = 1,287) were selected from the ARC nonword database (Rastle, Harrington, & Coltheart, 2002). The order of presentation of pre-stimulus tasks (words, nonwords, pseudohomophones) was randomized for each participant. The stimuli in the pre- and post-tasks were also counter-balanced across participants. Each list contained 40 letter strings, for a total of 240 letter strings (see Appendix A in online supporting information located at https://www.mitpressjournals.org/doi/suppl/10.1162/nol_a_00020).

#### Rapid naming task

A standard 4 × 9 array of letters (i.e., c, n, s, a, k, t) and digits (i.e., 2, 3, 4, 5, 7, 8) as per the Comprehensive Test of Phonological Processing (CTOPP; Wagner, Torgesen, Rashotte, & Pearson, 1999) was used. The following procedure was used to create the 4 × 9 array: a 6″ × 8″ grid was created that was partitioned into 36 cells. The six letters were randomly inserted into the array with the following restrictions: (1) no letter/digit was presented more than two times in a row, and (2) no letter/digit was presented twice in sequence (including controlling for a letter presented at the end of a row and the beginning of the next row). The letters/digits were presented in Calibri 68 pt. font on a Dell Vostro laptop, running Windows 7, with a screen resolution of 1366 × 768. Following this procedure, five unique arrays of letters and digits were created. Wagner et al. (1999) reported test–retest reliability for letters and digits in adults to be 0.86 and 0.90, respectively.

### Procedure

Upon arrival at the testing area, individuals were provided with a verbal explanation of the study, requested to read the terms of the study (with assistance if necessary), ask any questions, and sign the consent form. After consenting to the experiment, they were asked to complete a reading history questionnaire (Adult Reading History Questionnaire; see Parrila, Georgiou, & Corkett, 2007, for details) and a health evaluation form for screening. Participants then completed both the Word Identification and Word Attack tests (Woodcock & Johnson, 1989) to further characterize their overall reading abilities.

#### Pre-stimulation testing

Participants were seated in front of a monitor in the testing room for the administration of the pre-stimulus tests. One microphone was affixed between the eyebrow region with the microphone surface directed downwards and placed 10 cm away from the mouth, and a second free-standing microphone was placed directly in front of the mouth to record participant onset response time. The pre-stimulus tasks (the same as for post) included a fluency measure (rapid automatized naming (RAN) of digits and letters) and a reading measure (overt word naming of real words, nonwords, and pseudohomophones). The order of task presentation was randomized for each participant. All tasks were programmed in, and delivered through, E-Prime 3.0 software (Psychology Software Tools, Pittsburgh, PA, USA) using a standard Dell computer with a secondary monitor extension to avoid distracting the participant during the coding process. Participants were told that they would see some familiar and unfamiliar words on the screen, and their goal was to read aloud the words as quickly and accurately as possible as they appeared in the centre of the screen one by one. Halfway through the task, participants were offered a break, which typically lasted <1 min.

The examiner coded for accuracy of the responses by button-presses on the laptop (1 = correct pronunciation; 2 = incorrect; 3 = spoil). Voice recordings of the participants during the word reading task were collected via the program TF32, and were stored as WAV/RIFF audio files. Coding of accuracy was verified after data collection. For RAN tasks, participants were instructed to read the individual letters or digits as quickly and accurately as possible from left to right on the screen, from top to bottom of the page. Participants were given a practice trial of a single string of characters before being given the real task, which consisted of two 9 × 4 arrays (randomly selected from the five possible arrays). The examiner coded the RAN tasks by indicating number of errors made per array.

#### Stimulation delivery

**Figure 1.**
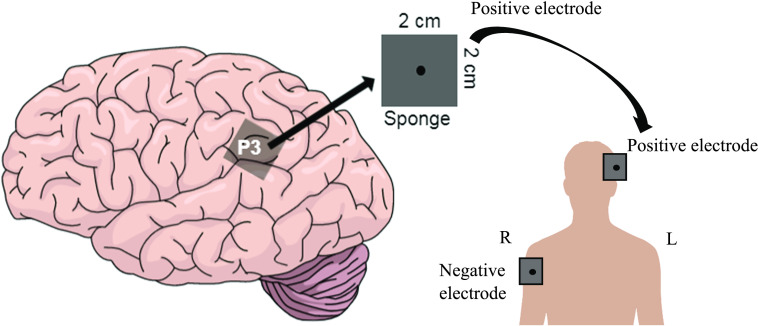
A representation of the positioning of the active/sham electrode. The region corresponding to P3 on the left hemisphere was targeted with a 2 cm × 2 cm positive electrode. The extracephalic negative electrode (same dimensions) was placed on the contralateral upper right arm.

After pre-stimulation testing, the left supramarginal gyrus region of the scalp at P3 in the 10–20 EEG system was prepared for stimulation. We measured the distance between the nasion and the inion and marked the halfway point corresponding to Cz. Subsequently, we measured 10% up from both nasion and inion to locate our Fpz and Oz points. Afterwards, we measured from tragus to tragus, and located the midway point at the intersection with the halfway point from the nasion to the inion (the true Cz). We then aligned this with the Fpz and Oz marks to determine their locations. The EEG cap was then fixed on the participant’s head, aligning it with Fpz and Oz to locate P3 on the scalp, and then removed. We prepared the P3 scalp region and the right shoulder by swabbing a 2 × 2 cm patch (dimensions of sponges used) with NuPrep gel to reduce skin impedance and pinning long hair out of the way to maximize conduction. We then affixed the anodal sponge electrode on the P3 region and placed the cathodal sponge electrode on the right upper arm/shoulder (in line with the armpit region) with a rubber band (Figure 1).

Approximately 7 mL of saline (0.9%; 36 g/4 L concentration) was used to wet both 2 × 2 cm sponge electrodes. Preceding the electrode placements, the stimulator was prepared to deliver 1.5 mA of current for 15 min corresponding to a current density of 0.375 mA/cm^2^. Subjects received either sham (*N* = 31; Impaired = 16; Skilled = 15) or anodal (*N* = 29; Skilled = 13; Impaired = 16) stimulation for 15 min from a Chattanooga Iontophoresis tDCS device (Chattanooga Group, Hixon, TN, USA). During anodal stimulation, 1.5 mA of current was applied to the scalp for 15 min, with 30 s for ramp-up and ramp-down time. Conversely, for the sham stimulation, participants received 30 s of ramp-up to provide the initial sensation of tingling and itching on the scalp for the purpose of blinding them to the identity of the tDCS condition. After 30 s, current was shut off. For the last thirty s of stimulation, current was then turned back on again and the device was shut off.

This protocol follows closely that of Turkeltaub et al. (2012) in the following ways: (1) an adult population, (2) application of 1.5 mA of current, (3) 15 min of stimulation concurrent with training, and (4) training at the level of a phonemic awareness (i.e., the ability to segment words into phonemes; see Turkeltaub et al., 2012, Figure 1, left side view for a representative current flow visual). In contrast to Turkeltaub, we used a smaller electrode size, which served to increase our current density to 0.375 mA/cm^2^ vs. 0.06 mA/cm^2^ and placement of our negative electrode on the contralateral upper right arm. In both conditions, participants were told that the stimulation could feel tingly or itchy but should never hurt, and if they ever felt discomfort the researcher would immediately discontinue the stimulation. No participants withdrew. The experimenter delivering the stimulation was not blind to the condition. However, scoring for the reading and rapid naming tasks was completed offline by blind experimenters.

#### Training protocol

Before turning stimulation on (for either anodal or sham), participants were seated in front of another computer monitor for a phoneme segmentation training task (see Appendix B in the online supporting information; https://apps.ankiweb.net/) containing 150 nonword stimuli. This remediation approach was chosen as the most promising reading programs focus on the small sound units (e.g., Eden et al., 2004; Serniclaes, Collet, & Sprenger-Charolles, 2015). Participants were instructed to segment the different nonwords according to the different sounds that they believed made up the word using the keyboard. Participants then completed a practice round of ten examples where they could ask questions for further explanation from the research assistant to ensure they fully understood the task before stimulation and the actual task began. Participants were immediately alerted whether their response was correct or incorrect. Incorrect answers were returned to the pool of stimuli and would show up again until they were correctly completed (at which point they would not reappear in the task). At the 15-min mark, the current was ramped down, regardless of whether or not the participants had completed segmentation of all possible nonwords in the training task. If participants completed the task earlier, they were instructed to quietly remain seated until the full dose of current was successfully delivered. This resulted in two individuals (skilled readers) who completed the task at 12 min and 13 min.

#### Post-stimulation testing

After the concurrent administration of the anodal/sham stimulation and the training segmentation task, the stimulation device was removed and participants were instructed to return to their original monitor to complete the final round of fluency and reading tasks. The microphone was affixed once more to their forehead, and tasks were presented in a randomized order identical to the method used in pre-stimulation testing. The post-stimulation testing occurred approximately 5 min after stimulation finished.

## RESULTS

All statistical tests were performed in SPSS 21. Dependent variables included mean reading speed for the rapid naming tasks and mean correct response time for the word/non-word/pseudohomophone task. Responses times <200 ms were considered outliers and removed prior to analysis. We used a series of mixed ANOVAs to test for the impact of tDCS and training on reading performance. Throughout the analysis process, we evaluated the extent to which each participant was an outlier (i.e., both across the entire sample and within each group). We did this for every variable. We also ensured that whenever an outlier was found, the analyses we ran were consistent both with and without the individual data point. When appropriate, we corrected for the violation of sphericity (*p* < 0.05; Mauchly’s test of sphericity, which compares equality between variances) using the Greenhouse Geisser Correction. When appropriate, we used Bonferroni corrections for *t* tests and Wilcoxon signed-rank tests to test pre–post differences on the reading and rapid naming measures (see Appendix C in the online supporting information for power calculations for the various analyses).

The difference in number of nonwords segmented during the training phase approached significance, with skilled readers going through 140.46 nonwords and impaired readers going through 128.9 nonwords, (*p* = 0.07). This difference was in the absence of a difference in the number of nonwords needing to be repeated (i.e., incorrectly segmented the first time), between skilled and impaired, 39.82 versus 40.1, respectively, *p* = 0.957.

### Reading of Regular Words, Pseudohomophones and Nonwords: Response Time

**Figure 2.**
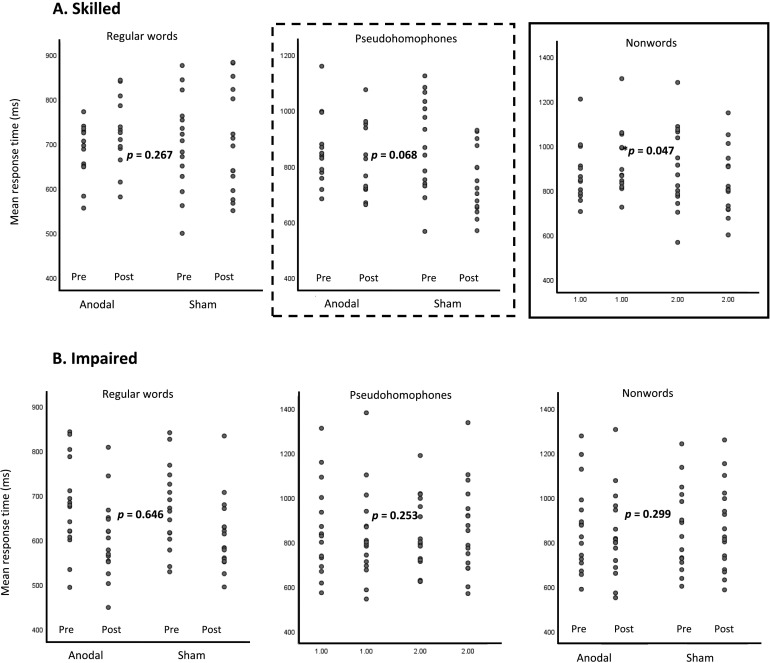
Mean correct response times, for each individual, as a function of stimulation condition and time for (A) Skilled readers (regular words, pseudohomophones, nonwords) and (B) Impaired readers (regular words, pseudohomophones, nonwords). Boxes indicate significant (*p* < 0.05) and marginally significant (*p* = 0.068) interactions in the 2 (Stimulation) × 2 (Time) ANOVA. No significant differences were found (pre vs. post) using the Wilcoxon signed-rank test.

A 2 (skilled vs. impaired) × 2 (anodal vs. sham) × 3 (regular words, pseudohompohones, nonwords) × 2 (pre vs. post) mixed ANOVA was run (see Table 2). The four-way interaction was significant, *F*(2, 110) = 4.702, *p* = 0.011. So, a series of 2 × 2 mixed ANOVAs were conducted separately for each reading group (skilled vs. impaired) with stimulation as the between-subjects factor and session time (pre-stimulation and post-stimulation) as the within-subject factor. For the skilled readers, there was no significant 2-way interaction for regular words (*p* = 0.267), a marginally significant 2-way interaction for the pseudohomophones (*p* = 0.068), and a significant 2-way interaction for the nonwords (*p* = 0.047). The follow-up Wilcoxon signed-rank tests, however, did not show any significant pre–post differences (see Figure 2A). For the impaired readers, there were no significant effects for regular words, pseudohomophones, or nonwords (see Figure 2B). Given the limited effects of the reading ability factor in each of the analyses, we also ran a subsequent set of analyses with this factor removed to determine if this increased the likelihood of finding a stimulation effect (see Supplementary Results and Appendix C in the online supporting information). The findings and interpretation did not change substantially, with no effects of anodal tDCS on reading performance or rapid naming.

**Table 2.** Mean correct response time (standard deviation) as a function of stimulation condition (anodal vs. sham), reading ability (skilled vs. impaired), and stimulus type (regular words [REG], pseudohomophones [PH], nonwords [NW])

		REG-pre	REG-post	PH-pre	PH-post	NW-pre	NW-post
Anodal	Skilled	683.47 (63.00)	725.05 (80.09)	856.46 (129.69)	916.59 (163.51)	877.67 (133.33)	930.09 (150.89)
	Impaired	676.84 (101.67)	684.47 (124.84)	855.84 (202.42)	822.36 (205.64)	868.47 (198.86)	836.05 (193.50)

Sham	Skilled	701.82 (108.79)	703.84 (122.08)	885.51 (171.54)	832.23 (153.70)	903.78 (191.12)	845.95 (157.13)
	Impaired	673.14 (92.97)	693.85 (112.76)	833.08 (166.49)	849.61 (199.33)	861.29 (185.26)	865.96 (192.54)

### Reading of Regular Words, Pseudohomophones, and Nonwords: Accuracy

A 2 (skilled vs. impaired) × 2 (anodal vs. sham) × 3 (regular word, pseudohomophone, nonword) × 2 (pre vs. post) mixed ANOVA was run (see Table 3). There was a significant main effect of stimulus type, *F*(1.272, 69.98) = 6.267, *p* = 0.009. Follow-up Bonferroni corrected *t* tests showed that regular words (84.4%; *SE* = 1.6) were significantly less accurate than pseudohomophones (87.1%; *SE* = 1.9; *p* = 0.018) and nonwords (86.9%; *SE* = 2.0; *p* = 0.015). No other effects were significant.

**Table 3.** Mean accuracy (standard deviation) as a function of stimulation condition (anodal vs. sham), reading ability (skilled vs. impaired), and stimulus type (regular words [REG], pseudohomophones [PH], nonwords [NW])

		REG-pre	REG-post	PH-pre	PH-post	NW-pre	NW-post
Anodal	Skilled	79.8 (20.9)	76.8 (26.6)	81.9 (22.1)	79.2 (29.7)	81.7 (21.7)	79.5 (28.9)
	Impaired	82.0 (14.7)	85.1 (6.8)	87.3 (18.1)	89.5 (12.6)	85.9 (19.1)	89.5 (9.3)

Sham	Skilled	84.2 (9.7)	87.0 (11.9)	86.0 (12.6)	89.8 (14.2)	86.4 (13.3)	89.7 (15.9)
	Impaired	91.5 (7.8)	90.1 (6.0)	92.1 (8.6)	90.2 (9.3)	94.1 (8.2)	90.6 (9.0)

### Rapid Naming of Letters and Digits: Response Time

**Figure 3.**
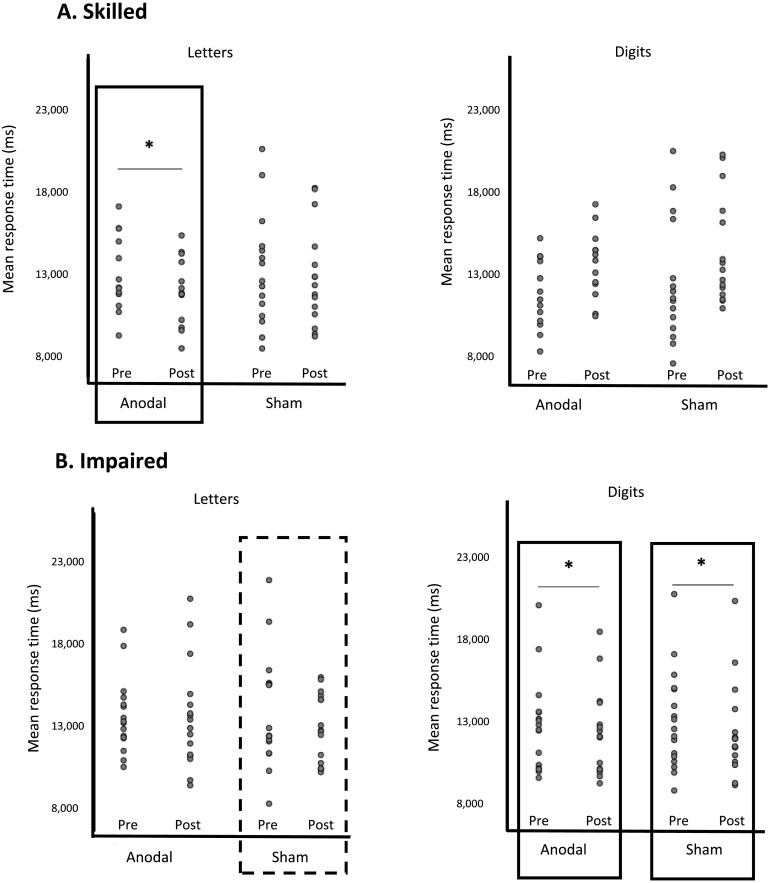
Mean response times for each participant, as a function of stimulation and time for (A) Skilled readers (letters and digits) and (B) Impaired readers (letters and digits). Boxes indicate significant (*p* < 0.05) difference on Wilcoxon signed-rank test. Dashed boxes indicate trend for significant (*p* = 0.088) difference on Wilcoxon signed-rank test.

A 2 (skilled vs. impaired) × 2 (anodal vs. sham) × 2 (letters vs. digits) × 2 (pre vs. post) mixed ANOVA was run (see Table 4). There was a significant main effect of time, indicating that responses before training were slower than afterwards (13,349 vs. 12,881 ms, respectively *F*(1, 56) = 11.58, *p* = 0.001. In addition, there was a main effect of stimulus type indicating that participants responded more quickly to digits than letters (12,760 vs. 13,470 ms), *F*(1, 56) = 25.32, *p* < 0.001 (Figure 3A and Figure 3B).

**Table 4.** Mean correct response time (standard deviation) as a function of stimulation condition (anodal vs. sham), reading ability (skilled vs. impaired), and rapid automatized naming (letters, digits)

		Letters-pre	Letters-post	Digits-pre	Digits-post
Anodal	Skilled	13,387.12 (2,182.91)	12,405.58 (1,956.82)	12,225.42 (1,985.27)	12,013.58 (2,281.53)
	Impaired	13,919.78 (2,139.14)	13,881.75 (3,025.82)	13,152.75 (2,670.99)	12,673.0 (2,445.52)

Sham	Skilled	13,586.67 (3,242.77)	13,245.57 (2,845.77)	12,993.83 (3,538.35)	12,911.70 (3,600.43)
	Impaired	14,147.06 (3,181.57)	13,188.09 (1,979.14)	13,376.56 (2,841.10)	12,728.53 (2,605.39)

### Rapid Naming of Letters and Digits: Accuracy

A 2 (skilled vs. impaired) × 2 (anodal vs. sham) × 2 (letters vs. digits) × 2 (pre vs. post) mixed ANOVA was run (see Table 5). There was a significant main effect of stimulus type, *F*(1, 56) = 28.74, *p* < 0.001. A follow-up paired samples *t* test showed that overall digits (99.2%; *SD* = 1.2) were more accurate than letters (97.6%; *SD* = 2.0), *p* < 0.001.

**Table 5.** Mean accuracy (standard deviation) as a function of stimulation condition (anodal vs. sham), reading ability (skilled vs. impaired), and rapid automatized naming (letters, digits)

		Letters-pre	Letters-post	Digits-pre	Digits-post
Anodal	Skilled	97.4 (2.7)	98.1 (2.1)	99.6 (1.0)	99.2 (1.8)
	Impaired	97.9 (1.9)	97.2 (3.2)	99.5 (1.1)	99.3 (1.9)

Sham	Skilled	97.4 (4.4)	98.9 (1.8)	99.4 (1.2)	98.9 (2.0)
	Impaired	96.9 (3.2)	96.2 (2.5)	98.4 (3.4)	97.9 (2.7)

## DISCUSSION

In this study, we investigated the effects of anodal tDCS, concurrently delivered alongside a computerized training protocol, on reading performance for skilled and impaired adult readers. While we found some effects of the computerized training protocol for impaired readers (i.e., faster rapid naming performance post training), we found weak-to-no evidence for an added effect of anodal stimulation on reading performance/speed for either skilled or impaired readers. Below, we discuss our findings within the context of current tDCS-reading literature, provide several considerations about the potential usefulness (or lack thereof) of tDCS as a neuromodulation tool to enhance/promote neuroplasticity in cognitive based tasks, and discuss the limitations of the tool.

Our (lack of) findings are in line with several previous studies that have sought to examine the potential of tDCS as a remediation tool (see Horvath, Forte, & Carter, 2015, for a meta-analysis). Consistent with the notion that tDCS is not efficacious in a healthy population on cognitive-based tasks, we did not find support for the role of anodal tDCS in enhancing reading performance in healthy young adults. We do note that our findings here are limited to single session tDCS effects. It does remain to be seen whether tDCS paired with skill-based training, on a repeated basis, and/or over a longer period of time, produces reliable and consistent effects that can be measurable in cognitive-based tasks. How best to implement such work, without reliable single session effects to begin with, needs to be carefully addressed. Single session data informs multisession data in several ways. For example, single session (and single electrode placement) data is important for understanding at what time point we can anticipate changes in behaviour to begin. At this point, the field is still ambiguous about whether we need to see effects in session 1, or session 2, or perhaps as long as in session 5. Such information is necessary to the literature on tDCS so researchers can make informed decisions about study design, participant recruitment, implementation, and cost/benefit ratios associated with sessions versus outcomes/gains. Single session data also provides information about what is potentially not working in the field. Without the careful documentation of single session findings, we as researchers could be spending a lot of time testing out some single session montages, tasks, and so forth, for which other researchers have already tested and established do not work, but which are absent from the literature. This missing literature has the potential to impede progress in the tDCS field, as we only have a limited spectrum of null versus significant findings to guide our future tDCS endeavours. Likely, there are many groups around the world that have run similar experiments to the one outlined here but have not published their non-significant findings for a variety of reasons. In an attempt to save future researchers some time, money and effort, the current study provides some guidance on what may not work for this particular population. In line with the recommendations of Horvath et al. (2015), we provide a comprehensive documentation of our measures, our results (both null and significant) and our data (i.e., means, standard deviations, etc.), in the hopes of facilitating future assessment of our findings within the larger context of additional tDCS studies.

To further complicate the matter, the effects of reading classification and remediation are quite varied. With respect to reading classification, the categorization of reading ability/disability is subjective—especially for adults who undoubtedly have developed compensatory strategies and/or trained extensively on a particular reading subskill (i.e., phonological awareness). For example, it is not uncommon to have individuals with an actual diagnosis of dyslexia who perform within typical limits on multiple reading tasks (Sela, Izzetoglu, Izzetoglu, & Onaral, 2012). Categorization of reading ability is a complicated process, and the findings of the current work are limited to the approach taken here (see collapsed analysis in the Supplement in the online supporting information). While the most promising remediation approaches focus on the small sound units (e.g., Eden et al., 2004; Serniclaes et al., 2015), these are most often effective at younger ages, but are still quite variable with responders and nonresponders present in all cohorts. Indeed, Costanzo et al. (2016b) reported no remediation effects for the children in their sham group (i.e., training alone) even though they were provided with 20-min cognitive training sessions, 3 times per week, for 6 weeks. For adults, training on phonemic awareness (the ability to segment words into phonemes; Serniclaes et al., 2015) is a common approach to reading remediation. This can include training on the small units of sound via word/nonword segmentation, phoneme deletion, and phoneme substitution tasks, just to name a few (Eden et al., 2004). Generalization to single word and nonword reading is ultimately a marker of more effective training approaches although such generalized findings are not often found (Eden et al., 2004). On the other hand, Turkeltaub et al. (2012), which the present study most closely resembles, reported real word reading improvements in below average reading adults following a single session tDCS plus training task (effect size approximately 0.34). More specifically, they applied anodal current to the left parietotemporal region, at 1.5 mA concurrently with a 15-min training task (either a phoneme perception task or a colour perception task; note that stimulation lasted 20 min, with the last 15 min concurrent with training). The extent to which the different effects reported in Turkeltaub et al. (2012) versus the current study are a result of the placement of the negative electrode (i.e., right homologue vs. right shoulder, respectively), type of remediation, type of outcome measures, and so forth are additional questions that need to be explored when considering tDCS plus training for reading remediation.

While we cannot be certain that the null effects reported with reading ability as a factor are not due to a lack of power, our findings are in line with a growing body of literature that reports limited effects of tDCS in adults. Further support for this notion is evident in our rapid naming outcome measurement, whereby we did have enough power to show generalized effects of training on rapid naming of letters and digits, but again in the presence of a null effect of stimulation on this outcome measure. As such, we interpret the null effects as a reflection of the limitations of tDCS as a neuromodulation tool for use in an adult population and with a cognitive task, such as reading. Adult brains are far less plastic than paediatric brains, which may be one reason that findings in the paediatric literature appear more promising (Costanzo et al., 2016a). Of course, we know that adult brains can, and do, still change in response to treatment/remediation; however, the nature of a single dose of tDCS (at 1.5mA, over 15 min, and with a concurrent training task, as was administered here) may not be the most effective approach, in comparison to transcranial magnetic stimulation for example, that actually induces action potentials (although see Costanzo, Menghini, Caltagirone, Oliveri, & Vicari, 2012, who showed that transcranial magnetic stimulation of the temporoparietal region improved nonword accuracy but not fluency). With respect to the latter, reading is a cognitive task that recruits an entire network of brain regions, regardless of one’s reading ability. Reading, in general, involves frontal, occipitotemporal, and temporoparietal regions (Richlan et al., 2009; Sandak et al., 2004). It is quite conceivable that stimulation to a single region in a network is not sufficient to upset the natural state of the system. Future work that explores multiple left hemisphere stimulation centres concurrently would be needed to fully test this claim.

There has been a bias toward bipolar montages in the reading space, albeit in the absence of systematic supporting evidence, and thus additional work that focuses on the optimal montage placement in the reading domain is still needed. With respect to the current work, we cannot be certain that the montage utilized was optimal for the current task and/or training paradigm, even in light of recent simulation work that provided evidence for a unipolar montage. More specifically, Bhattacharjee et al. (2019) recently explored the effect of various bipolar (i.e., positive and negative electrodes placed on the head) and unipolar (i.e., the positive electrode on the head and the negative electrode placed extracephalically) montages on dorsally versus ventrally mediated reading pathways. Indeed, one of the montages proving to be optimal (i.e., simulated maximal targeted activity) was a unipolar montage with the positive electrode placed on the left temporal region (i.e., TP7) and the negative electrode placed extracephalically (i.e., on the neck). While unipolar montages (i.e., one electrode on the head and the other placed extracephalically) can lead to greater focal distribution of current as compared to bipolar montages, where it is more difficult to exclude the effect of the negative electrode (Im, Park, Shim, Chang, & Kim, 2012), there is a strong bias toward bipolar montages in the reading space (e.g., Thomson et al., 2015; Turkeltaub et al., 2012). Hence, the mixed tDCS-reading findings are likely resulting from several subjective factors including montage (unipolar vs. bipolar vs. multi-electrode arrays), electrode placement, training paradigm, and task of interest, just to name a few. The present work is limited by these same factors. Here, we used a unipolar approach, with a P3 positive electrode placement, a phoneme segmentation training task, and an overt single-word reading paradigm. While this montage is important to report on, as the brain stimulation field works to provide a systematic approach to filling in the gaps in the tDCS-reading literature, ultimately we cannot be certain to what extent the null effects reported here indicate the limits of tDCS as a tool or the limits of tDCS methodology in general.

Although not the focus of the current work, we did note some interesting trends with respect to the computerized segmentation training, which was based on research pertaining to targeting basic reading processes such as phoneme segmentation (Snowling & Hulme, 2012; Alexander & Slinger-Constant, 2004). Specifically, we saw small training effects for the rapid naming tasks. The computerized program we used here provided participants with feedback (accurate, inaccurate, and correct response) on every trial. In addition, incorrect stimuli were rerandomized back into the larger list for participants to attempt again until they were successful. Our approach to skill-based training plus single session stimulation was similar to the study by Costanzo et al. (2016a), who demonstrated that training children with dyslexia, on both reading aloud and a silent phonics task, alongside single session anodal tDCS sessions, was able to improve accuracy for low frequency words, as well as nonword reading speed. While we did not find evidence for such effects of anodal tDCS to generalize to an adult population of skilled and impaired readers, the skill-based training may be one avenue of future work to consider. For example, perhaps a training task such as overt phoneme segmentation, which would be more similar to the overt outcome measures, might provide an optimal match between modulated training and measured outcome with respect to the impaired group.

Our results do not readily align with prior studies that suggest anodal stimulation of the left temporoparietal regions can improve reading ability (Younger et al., 2016; Turkeltaub et al., 2012; Costanzo et al., 2016a, 2016b; Heth & Lavidor, 2015). Given that the temporoparietal junction is a large region, consisting of the posterior superior temporal gyrus, the inferior supramarginal gyrus, and the angular gyrus (Ramus, 2004), and that our electrode size (2 × 2 cm) was wide enough to deliver current across these regions, we cannot be certain that spreading effects did not contribute to our null findings (although see Costanzo et al., 2016a, who used a 5 × 5 cm electrode and reported positive effects). Notably, it is still somewhat unclear to what extent the current travels beyond the targeted region(s) both peripherally and centrally to deeper brain structures (although see Opitz, Paulus, Will, Antunes, & Thielscher, 2015, and Thielscher, Antunes, & Saturnino, 2015, for discussions on how to model the effects of neuromodulation). Similar to our point above about the larger reading network, it is possible that the balance between cortical inhibition and excitation was disrupted in some manner, leading to less than optimal results (Krause & Kadosh, 2013). Computational models of current flow are becoming increasing popular in the literature to provide a guide as to where electrode placement may be ideal (i.e., see Turkeltaub et al., 2012, Figure 1 left side view for a current flow model; see also Bhattacharjee et al., 2019, for a simulation study of various montages); however, the challenges with (1) the extent to which such models accurately represent current flow and (2) the individual variability that impacts the many variables used to create computational current models (i.e., skin, bone, muscle, cerebral spinal fluid, gyri and sulci folding, and so forth; Bikson, Rahman, & Datta, 2012; Sadleir, Vannorsdall, Schretlen, & Gordon, 2010) remain unclear as to their impact on subsequent outcomes. For example, while a unipolar montage was one of the ideal montages presented in Bhattacharjee et al. (2019), such an approach did not produce significant effects in the current work. Ultimately, conclusions about potential spreading effects are difficult to tease apart without considerably more complex setups (i.e., multisites), more focal stimulation (i.e., an electrode size on the order of millimetres), and paired brain imaging approaches (i.e., fMRI).

### General Discussion

While it is attractive to think that excitation or inhibition of one brain region can produce immediate, marked, and potentially long-lasting benefits, the inconsistent findings with tDCS indicates that we *must* be cautious in perpetuating such gross claims. Neural circuits are intimately connected to each other and modulations in one region undoubtedly influence other regions. A delicate balance between inhibition and excitation needs to be maintained for optimal behavioural performance, whether that be typical or compensatory in nature, depending on the population of study (e.g., skilled vs. impaired readers). Further, there is much evidence for individual variation in normal brain states (Graves et al., 2014), making it even more difficult to ascertain if excitatory or inhibitory stimulation is what is needed to change nonoptimal levels. While it is well accepted that left hemisphere regions are impaired, and typically hypoactive, in those with reading disabilities, it is also documented that right hemisphere regions are recruited to compensate for the dysfunction (Vandermosten, Boets, Wouters, & Ghesquière, 2012; Eden et al., 2004), in effect producing a different state of normal for the impaired reader. Given that we do not see what immediately occurs at a molecular level (Krause & Kadosh, 2013) following tDCS in humans also makes our approaches and assumptions about stimulation protocols and effects somewhat unwieldy. Therefore, it remains a question as to how tDCS can impact the natural compensatory mechanisms in nontypical populations.

Lastly, while we are aware of how behavioural training can affect outcomes, we do not know what regions tDCS is affecting in the neural networks. Although single sessions of tDCS are reported to have transient aftereffects and fade away after a few min to an hour (Brunoni et al., 2012), the reality is that most researchers utilize tDCS in the hopes of improving long-term outcomes. While it is easy to say that we have not impacted certain behavioural performances (i.e., reading), we cannot truly be sure that the stimulation did not affect anything else. This brings us to the open-ended question: Is tDCS really noninvasive if it has the potential to cause lasting changes that we might not yet understand the full consequences of?

### Conclusion

Given the many challenges faced in the assessment, diagnosis, and treatment of developmental dyslexia, novel approaches to remediation have become increasingly explored, namely neurostimulation tools (e.g., tDCS; Turkeltaub et al., 2012; Younger et al., 2016; Heth & Lavidor, 2015; Costanzo et al., 2016a, 2016b). While findings in the paediatric literature appear promising, the effects of tDCS in adult populations warrant a more pessimistic perspective. Here, we found no evidence for the effects of anodal tDCS, concurrently delivered alongside a computerized training protocol, on reading performance for skilled and impaired adult readers. The extent to which our null findings are (1) evidence against tDCS effectiveness, (2) a result of our methodological design (i.e., montage, training paradigm, task performance, etc.), and/or (3) some combination of these factors, remains to be seen. Ultimately, such findings have implications for our understanding of the complex nature of the reading network, and our inability to disrupt it, and more broadly raise the question about continued use of tDCS in healthy adult populations.

## ACKNOWLEDGMENTS

The work outlined in this paper was partially funded by an Natural Sciences and Engineering Research Council of Canada operating grant to author Jacqueline Cummine.

## FUNDING INFORMATION

Jacqueline Cummine, Natural Sciences and Engineering Research Council of Canada (http://dx.doi.org/10.13039/501100000038), Award ID: 2018-03992.

## AUTHOR CONTRIBUTIONS

Jacqueline Cummine and Joseph T. Devlin were responsible for experimental design, data analysis, and interpretation; and manuscript development and revision. Miya Villarena was responsible for data collection, analysis, and manuscript revision. Taylor Onysyk was responsible for data collection and manuscript revision.
